# *N*-[2-(1,3-Benzodioxol-5-yl)eth­yl]-2-chloro­acetamide

**DOI:** 10.1107/S1600536808014232

**Published:** 2008-05-21

**Authors:** Hui-Chao Dong

**Affiliations:** a Henan Provincial Key Laboratory of Surface & Interface Science, Zhengzhou University of Light Industry, Zhengzhou 450002, People’s Republic of China

## Abstract

The title compound, C_11_H_12_ClNO_3_, crystallizes with two independent mol­ecules in the asymmetric unit. Inter­molecular N—H⋯O hydrogen bonds link the mol­ecules related by translation along the *b* axis into two independent hydrogen-bonded chains. The crystal studied exhibited inversion twinning.

## Related literature

For the crystal structures of related compounds, see: Kuehne *et al.* (1988 ▶). For details of the aplication of *N*-(2-benzo[1,3]dioxol-5-yl-ethyl)-2-chloro-acetamide, see: Bernhard & Snieckus (1971 ▶); Ma *et al.* (2006 ▶). For bond-length data, see Allen *et al.* (1987 ▶).
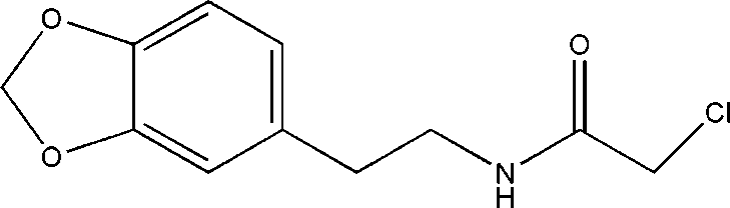


## Experimental

### 

#### Crystal data

C_11_H_12_ClNO_3_
*M* *_r_* = 241.67
Orthorhombic, 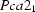

*a* = 14.429 (3) Å
*b* = 5.1258 (10) Å
*c* = 30.679 (6) Å
*V* = 2269.1 (8) Å^3^
*Z* = 8
Mo *K*α radiation
μ = 0.33 mm^−1^
*T* = 293 (2) K
0.20 × 0.12 × 0.09 mm

#### Data collection

Rigaku R-AXIS RAPID IP area-detector diffractometer
Absorption correction: multi-scan (*ABSCOR*; Higashi, 1995 ▶) *T*_min_ = 0.937, *T*_max_ = 0.971
15836 measured reflections
3949 independent reflections
2946 reflections with *I* > 2σ(*I*)
*R*_int_ = 0.029

#### Refinement

*R*[*F* ^2^ > 2σ(*F* ^2^)] = 0.035
*wR*(*F* ^2^) = 0.110
*S* = 1.13
3949 reflections
290 parameters
1 restraint
H-atom parameters constrained
Δρ_max_ = 0.34 e Å^−3^
Δρ_min_ = −0.34 e Å^−3^
Absolute structure: Flack (1983 ▶), 1304 Friedel pairs
Flack parameter: 0.47 (8)

### 

Data collection: *RAPID-AUTO* (Rigaku, 2004 ▶); cell refinement: *RAPID-AUTO*; data reduction: *RAPID-AUTO*; program(s) used to solve structure: *SHELXTL* (Sheldrick, 2008 ▶); program(s) used to refine structure: *SHELXTL*; molecular graphics: *SHELXTL*; software used to prepare material for publication: *SHELXTL*.

## Supplementary Material

Crystal structure: contains datablocks I, global. DOI: 10.1107/S1600536808014232/cv2412sup1.cif

Structure factors: contains datablocks I. DOI: 10.1107/S1600536808014232/cv2412Isup2.hkl

Additional supplementary materials:  crystallographic information; 3D view; checkCIF report

## Figures and Tables

**Table 1:** Hydrogen-bond geometry (Å, °)

*D*—H⋯*A*	*D*—H	H⋯*A*	*D*⋯*A*	*D*—H⋯*A*
N1—H1*A*⋯O3^i^	0.86	2.18	2.894 (4)	140
N2—H2*B*⋯O6^ii^	0.86	2.18	2.894 (4)	140

Symmetry codes: (i) 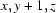; (ii) 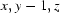.

**Fig. 1.**
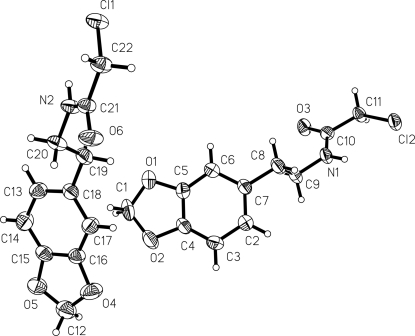
The content of asymmetric unit of (I), with atomic labels and 40% probability displacement ellipsoids for non-H atoms.

## supplementary crystallographic information

### Comment

The title compound (I) is an important intermediate for the synthesis of3, 4-dihydroisoquinoline and some other heterocyclic compounds (Bernhard & Snieckus, 1971; Ma *et al.*, 2006). In this paper, we  report its crystal structure.

Compound (I) crystallizes with two independent molecules in the non-centrosymmetric unit cell (Fig. 1). All bond lengths and angles in (I) are normal (Allen *et al.*, 1987) and in a good agreement with those reported previously (Kuehne *et al.*, 1988). The intermolecular N—H···O hydrogen bonds (Table 1) link the molecules related by translation along *b* axis into two independent hydrogen-bonded chains.

### Experimental

2-Benzo[1,3]dioxol-5-yl-ethylamine (20 mmol) was dissolved in CH_2_Cl_2_, and K_2_CO_3_ (30 mmol) was added, then chloroacetyl chloride (20 mmol) was added during 30 min at 273 K. After 2 h standing at room temperature, the solution was washed with water, the organic layer was separated, dried with Na_2_SO_4_ and evaporated to obtain the primary product. The pure product was isolated by recrystallization from ethyl acetate (1.50 g, 68%). Single crystals suitable for X-ray measurements were obtained by recrystallization from ethyl acetate at room temperature.

### Refinement

H atoms were positioned geometrically and refined using a riding model, with C—H = 0.93 or 0.97 Å, N—H=0.86Å and with *U*_iso_(H) = 1.2 times *U*_eq_(C, N).

### Crystal data

**Table:**

C_11_H_12_ClNO_3_	*F*_000_ = 1008
*M**_r_* = 241.67	*D*_x_ = 1.415 Mg m^−^^3^
Orthorhombic, *P**c**a*2_1_	Mo *K*α radiation λ = 0.71073 Å
Hall symbol: P 2c -2ac	Cell parameters from 2501 reflections
*a* = 14.429 (3) Å	θ = 2.3–25.1º
*b* = 5.1258 (10) Å	µ = 0.33 mm^−^^1^
*c* = 30.679 (6) Å	*T* = 293 (2) K
*V* = 2269.1 (8) Å^3^	Needle, colourless
*Z* = 8	0.20 × 0.12 × 0.09 mm

### Data collection

**Table:**

Rigaku R-AXIS RAPID IP area-detector diffractometer	3949 independent reflections
Radiation source: Rotating Anode	2946 reflections with *I* > 2σ(*I*)
Monochromator: graphite	*R*_int_ = 0.029
*T* = 293(2) K	θ_max_ = 25.0º
ω oscillation scans	θ_min_ = 3.1º
Absorption correction: multi-scan(ABSCOR; Higashi, 1995)	*h* = −17→17
*T*_min_ = 0.937, *T*_max_ = 0.971	*k* = −5→6
15836 measured reflections	*l* = −36→36

### Refinement

**Table:**

Refinement on *F*^2^	Hydrogen site location: inferred from neighbouring sites
Least-squares matrix: full	H-atom parameters constrained
*R*[*F*^2^ > 2σ(*F*^2^)] = 0.035	*w* = 1/[σ^2^(*F*_o_^2^) + (0.0541*P*)^2^ + 0.2974*P*] where *P* = (*F*_o_^2^ + 2*F*_c_^2^)/3
*wR*(*F*^2^) = 0.110	(Δ/σ)_max_ = 0.001
*S* = 1.13	Δρ_max_ = 0.34 e Å^−^^3^
3949 reflections	Δρ_min_ = −0.34 e Å^−^^3^
290 parameters	Extinction correction: SHELXTL (Sheldrick, 2008), Fc^*^=kFc[1+0.001xFc^2^λ^3^/sin(2θ)]^-1/4^
1 restraint	Extinction coefficient: 0.0035 (6)
Primary atom site location: structure-invariant direct methods	Absolute structure: Flack (1983), 1304 Friedel pairs
Secondary atom site location: difference Fourier map	Flack parameter: 0.47 (8)

### Special details

**Table:**

Geometry. All e.s.d.'s (except the e.s.d. in the dihedral angle between two l.s. planes) are estimated using the full covariance matrix. The cell e.s.d.'s are taken into account individually in the estimation of e.s.d.'s in distances, angles and torsion angles; correlations between e.s.d.'s in cell parameters are only used when they are defined by crystal symmetry. An approximate (isotropic) treatment of cell e.s.d.'s is used for estimating e.s.d.'s involving l.s. planes.
Refinement. Refinement of *F*^2^ against ALL reflections. The weighted *R*-factor *wR* and goodness of fit *S* are based on *F*^2^, conventional *R*-factors *R* are based on *F*, with *F* set to zero for negative *F*^2^. The threshold expression of *F*^2^ > σ(*F*^2^) is used only for calculating *R*-factors(gt) *etc*. and is not relevant to the choice of reflections for refinement. *R*-factors based on *F*^2^ are statistically about twice as large as those based on *F*, and *R*- factors based on ALL data will be even larger.

### Fractional atomic coordinates and isotropic or equivalent isotropic displacement parameters (Å^2^)

**Table:**

	*x*	*y*	*z*	*U*_iso_*/*U*_eq_	
Cl1	0.80437 (6)	0.77974 (17)	0.26938 (4)	0.0660 (3)	
Cl2	1.04831 (6)	0.72284 (17)	0.70767 (4)	0.0658 (3)	
O1	0.5138 (3)	0.4037 (8)	0.51198 (14)	0.1064 (13)	
O2	0.4074 (2)	0.7028 (7)	0.53628 (11)	0.0873 (10)	
O3	0.86302 (18)	0.1950 (5)	0.67866 (11)	0.0726 (8)	
O4	0.2640 (2)	1.1064 (7)	0.46419 (14)	0.1001 (12)	
O5	0.1615 (2)	0.7930 (7)	0.43980 (11)	0.0826 (9)	
O6	0.61927 (18)	1.3095 (5)	0.29844 (12)	0.0725 (8)	
N1	0.8579 (2)	0.6316 (6)	0.67326 (12)	0.0569 (8)	
H1A	0.8872	0.7749	0.6780	0.068*	
N2	0.6139 (2)	0.8733 (6)	0.30282 (13)	0.0567 (9)	
H2B	0.6428	0.7294	0.2980	0.068*	
C1	0.4217 (4)	0.4861 (11)	0.5078 (3)	0.086 (2)	
H1B	0.3799	0.3450	0.5154	0.103*	
H1C	0.4093	0.5371	0.4779	0.103*	
C2	0.6047 (3)	0.9174 (9)	0.60261 (15)	0.0694 (11)	
H2A	0.6226	1.0428	0.6228	0.083*	
C3	0.5145 (3)	0.9204 (10)	0.58685 (18)	0.0766 (12)	
H3A	0.4720	1.0442	0.5964	0.092*	
C4	0.4904 (3)	0.7377 (8)	0.55716 (14)	0.0607 (10)	
C5	0.5532 (3)	0.5584 (11)	0.5434 (2)	0.0649 (13)	
C6	0.6420 (3)	0.5492 (9)	0.5578 (2)	0.0681 (14)	
H6A	0.6836	0.4258	0.5473	0.082*	
C7	0.6685 (2)	0.7335 (7)	0.58913 (13)	0.0558 (9)	
C8	0.7652 (2)	0.7271 (8)	0.60849 (14)	0.0671 (11)	
H8A	0.8034	0.6082	0.5917	0.080*	
H8B	0.7925	0.8996	0.6064	0.080*	
C9	0.7648 (3)	0.6432 (7)	0.65481 (14)	0.0605 (10)	
H9A	0.7277	0.7642	0.6717	0.073*	
H9B	0.7363	0.4723	0.6569	0.073*	
C10	0.8985 (3)	0.4085 (7)	0.68301 (15)	0.0539 (10)	
C11	0.9951 (2)	0.4136 (7)	0.70259 (15)	0.0613 (10)	
H11A	1.0348	0.3041	0.6848	0.074*	
H11B	0.9920	0.3352	0.7313	0.074*	
C12	0.1728 (5)	1.0074 (11)	0.4679 (3)	0.088 (2)	
H12A	0.1611	0.9539	0.4977	0.105*	
H12B	0.1285	1.1426	0.4603	0.105*	
C13	0.3619 (3)	0.5967 (9)	0.37410 (14)	0.0662 (11)	
H13A	0.3813	0.4735	0.3538	0.079*	
C14	0.2717 (3)	0.5835 (10)	0.38929 (18)	0.0748 (12)	
H14A	0.2307	0.4562	0.3795	0.090*	
C15	0.2458 (3)	0.7656 (8)	0.41918 (13)	0.0605 (10)	
C16	0.3067 (3)	0.9502 (10)	0.43397 (19)	0.0592 (12)	
C17	0.3958 (3)	0.9658 (9)	0.4176 (2)	0.0668 (15)	
H17A	0.4359	1.0969	0.4267	0.080*	
C18	0.4239 (3)	0.7821 (7)	0.38744 (12)	0.0560 (9)	
C19	0.5200 (3)	0.7908 (9)	0.36873 (14)	0.0670 (11)	
H19A	0.5564	0.9174	0.3848	0.080*	
H19B	0.5489	0.6214	0.3724	0.080*	
C20	0.5208 (2)	0.8615 (7)	0.32136 (14)	0.0602 (10)	
H20A	0.4848	0.7339	0.3053	0.072*	
H20B	0.4912	1.0299	0.3177	0.072*	
C21	0.6554 (2)	1.0929 (7)	0.29311 (13)	0.0506 (9)	
C22	0.7510 (3)	1.0888 (7)	0.27470 (15)	0.0627 (10)	
H22A	0.7901	1.1967	0.2930	0.075*	
H22B	0.7491	1.1696	0.2461	0.075*	

### Atomic displacement parameters (Å^2^)

**Table:**

	*U*^11^	*U*^22^	*U*^33^	*U*^12^	*U*^13^	*U*^23^
Cl1	0.0492 (5)	0.0504 (5)	0.0983 (7)	0.0069 (4)	0.0175 (5)	0.0016 (6)
Cl2	0.0504 (5)	0.0516 (5)	0.0956 (7)	−0.0081 (4)	−0.0166 (5)	0.0014 (6)
O1	0.076 (2)	0.131 (3)	0.112 (3)	0.006 (2)	−0.031 (2)	−0.054 (3)
O2	0.0487 (16)	0.124 (3)	0.089 (2)	0.0023 (16)	−0.0162 (16)	0.001 (2)
O3	0.0640 (17)	0.0391 (13)	0.115 (2)	−0.0078 (11)	−0.0205 (16)	−0.0023 (16)
O4	0.075 (2)	0.103 (2)	0.122 (3)	−0.007 (2)	0.037 (2)	−0.041 (3)
O5	0.0531 (16)	0.107 (2)	0.087 (2)	−0.0059 (15)	0.0207 (16)	−0.0081 (19)
O6	0.0626 (17)	0.0394 (13)	0.115 (2)	0.0078 (11)	0.0219 (16)	−0.0040 (16)
N1	0.0437 (16)	0.0416 (16)	0.085 (2)	−0.0045 (13)	−0.0175 (16)	0.0032 (17)
N2	0.0424 (16)	0.0411 (17)	0.087 (3)	0.0053 (13)	0.0127 (16)	−0.0041 (17)
C1	0.066 (3)	0.122 (5)	0.069 (5)	−0.019 (3)	−0.030 (4)	0.005 (3)
C2	0.059 (2)	0.075 (3)	0.074 (3)	0.006 (2)	−0.009 (2)	−0.012 (3)
C3	0.059 (3)	0.090 (3)	0.080 (3)	0.026 (2)	−0.005 (3)	−0.013 (3)
C4	0.042 (2)	0.081 (3)	0.058 (2)	−0.0002 (19)	−0.0040 (17)	0.007 (2)
C5	0.055 (3)	0.072 (2)	0.067 (3)	−0.007 (2)	−0.005 (2)	−0.010 (3)
C6	0.053 (3)	0.078 (3)	0.073 (4)	0.009 (2)	−0.004 (2)	−0.010 (3)
C7	0.0438 (18)	0.068 (2)	0.056 (2)	−0.0028 (17)	−0.0025 (17)	0.004 (2)
C8	0.042 (2)	0.093 (3)	0.067 (3)	−0.0042 (18)	−0.0033 (17)	−0.001 (2)
C9	0.040 (2)	0.057 (2)	0.085 (3)	−0.0035 (15)	−0.0128 (17)	0.007 (2)
C10	0.052 (2)	0.039 (2)	0.071 (3)	−0.0008 (17)	0.001 (2)	−0.001 (2)
C11	0.0459 (19)	0.0435 (19)	0.094 (3)	0.0010 (15)	−0.016 (2)	0.005 (2)
C12	0.072 (4)	0.103 (5)	0.087 (6)	0.010 (2)	0.007 (4)	0.003 (3)
C13	0.061 (3)	0.069 (3)	0.068 (3)	−0.005 (2)	0.013 (2)	−0.005 (3)
C14	0.065 (3)	0.086 (3)	0.074 (3)	−0.025 (2)	0.012 (2)	−0.008 (3)
C15	0.046 (2)	0.075 (2)	0.061 (3)	0.0012 (19)	0.0091 (17)	0.010 (2)
C16	0.053 (3)	0.067 (2)	0.058 (3)	0.003 (2)	0.009 (2)	−0.001 (2)
C17	0.057 (3)	0.074 (3)	0.070 (4)	−0.011 (2)	0.012 (3)	−0.012 (2)
C18	0.049 (2)	0.066 (2)	0.054 (2)	0.0020 (17)	0.0007 (18)	0.0051 (19)
C19	0.044 (2)	0.088 (3)	0.068 (3)	0.001 (2)	0.0021 (17)	−0.003 (2)
C20	0.040 (2)	0.060 (2)	0.080 (3)	0.0049 (16)	0.0119 (17)	0.006 (2)
C21	0.0473 (19)	0.038 (2)	0.067 (2)	0.0004 (15)	0.0091 (19)	−0.002 (2)
C22	0.054 (2)	0.0424 (19)	0.092 (3)	0.0005 (16)	0.015 (2)	−0.003 (2)

### Geometric parameters (Å, °)

**Table:**

Cl1—C22	1.769 (4)	C7—C8	1.517 (5)
Cl2—C11	1.768 (4)	C8—C9	1.485 (6)
O1—C5	1.372 (7)	C8—H8A	0.9700
O1—C1	1.401 (7)	C8—H8B	0.9700
O2—C4	1.370 (5)	C9—H9A	0.9700
O2—C1	1.428 (7)	C9—H9B	0.9700
O3—C10	1.216 (4)	C10—C11	1.518 (5)
O4—C16	1.371 (6)	C11—H11A	0.9700
O4—C12	1.416 (7)	C11—H11B	0.9700
O5—C15	1.378 (5)	C12—H12A	0.9700
O5—C12	1.405 (7)	C12—H12B	0.9700
O6—C21	1.237 (4)	C13—C18	1.367 (6)
N1—C10	1.319 (5)	C13—C14	1.384 (6)
N1—C9	1.458 (4)	C13—H13A	0.9300
N1—H1A	0.8600	C14—C15	1.361 (6)
N2—C21	1.309 (5)	C14—H14A	0.9300
N2—C20	1.459 (4)	C15—C16	1.368 (6)
N2—H2B	0.8600	C16—C17	1.383 (6)
C1—H1B	0.9700	C17—C18	1.381 (6)
C1—H1C	0.9700	C17—H17A	0.9300
C2—C7	1.381 (6)	C18—C19	1.501 (5)
C2—C3	1.388 (6)	C19—C20	1.498 (6)
C2—H2A	0.9300	C19—H19A	0.9700
C3—C4	1.352 (7)	C19—H19B	0.9700
C3—H3A	0.9300	C20—H20A	0.9700
C4—C5	1.358 (7)	C20—H20B	0.9700
C5—C6	1.355 (6)	C21—C22	1.491 (5)
C6—C7	1.401 (7)	C22—H22A	0.9700
C6—H6A	0.9300	C22—H22B	0.9700
			
C5—O1—C1	106.5 (4)	C10—C11—H11A	108.1
C4—O2—C1	105.1 (4)	Cl2—C11—H11A	108.1
C16—O4—C12	105.2 (4)	C10—C11—H11B	108.1
C15—O5—C12	105.0 (4)	Cl2—C11—H11B	108.1
C10—N1—C9	122.2 (3)	H11A—C11—H11B	107.3
C10—N1—H1A	118.9	O5—C12—O4	109.8 (6)
C9—N1—H1A	118.9	O5—C12—H12A	109.7
C21—N2—C20	123.0 (3)	O4—C12—H12A	109.7
C21—N2—H2B	118.5	O5—C12—H12B	109.7
C20—N2—H2B	118.5	O4—C12—H12B	109.7
O1—C1—O2	108.4 (5)	H12A—C12—H12B	108.2
O1—C1—H1B	110.0	C18—C13—C14	123.2 (4)
O2—C1—H1B	110.0	C18—C13—H13A	118.4
O1—C1—H1C	110.0	C14—C13—H13A	118.4
O2—C1—H1C	110.0	C15—C14—C13	116.8 (4)
H1B—C1—H1C	108.4	C15—C14—H14A	121.6
C7—C2—C3	121.8 (4)	C13—C14—H14A	121.6
C7—C2—H2A	119.1	C14—C15—C16	121.4 (4)
C3—C2—H2A	119.1	C14—C15—O5	128.4 (4)
C4—C3—C2	117.9 (4)	C16—C15—O5	110.1 (4)
C4—C3—H3A	121.0	C15—C16—O4	109.9 (4)
C2—C3—H3A	121.0	C15—C16—C17	121.1 (5)
C3—C4—C5	120.4 (4)	O4—C16—C17	129.0 (5)
C3—C4—O2	129.1 (4)	C18—C17—C16	118.5 (4)
C5—C4—O2	110.5 (4)	C18—C17—H17A	120.8
C6—C5—C4	123.6 (5)	C16—C17—H17A	120.8
C6—C5—O1	126.9 (5)	C13—C18—C17	118.9 (4)
C4—C5—O1	109.4 (4)	C13—C18—C19	120.7 (4)
C5—C6—C7	117.2 (5)	C17—C18—C19	120.4 (4)
C5—C6—H6A	121.4	C20—C19—C18	112.7 (3)
C7—C6—H6A	121.4	C20—C19—H19A	109.0
C2—C7—C6	119.0 (4)	C18—C19—H19A	109.0
C2—C7—C8	120.7 (4)	C20—C19—H19B	109.0
C6—C7—C8	120.3 (4)	C18—C19—H19B	109.0
C9—C8—C7	112.2 (3)	H19A—C19—H19B	107.8
C9—C8—H8A	109.2	N2—C20—C19	113.3 (3)
C7—C8—H8A	109.2	N2—C20—H20A	108.9
C9—C8—H8B	109.2	C19—C20—H20A	108.9
C7—C8—H8B	109.2	N2—C20—H20B	108.9
H8A—C8—H8B	107.9	C19—C20—H20B	108.9
N1—C9—C8	112.4 (3)	H20A—C20—H20B	107.7
N1—C9—H9A	109.1	O6—C21—N2	123.3 (3)
C8—C9—H9A	109.1	O6—C21—C22	116.9 (3)
N1—C9—H9B	109.1	N2—C21—C22	119.8 (3)
C8—C9—H9B	109.1	C21—C22—Cl1	116.8 (3)
H9A—C9—H9B	107.9	C21—C22—H22A	108.1
O3—C10—N1	124.7 (4)	Cl1—C22—H22A	108.1
O3—C10—C11	116.4 (3)	C21—C22—H22B	108.1
N1—C10—C11	118.9 (3)	Cl1—C22—H22B	108.1
C10—C11—Cl2	116.7 (3)	H22A—C22—H22B	107.3
			
C5—O1—C1—O2	2.5 (7)	C15—O5—C12—O4	−0.5 (7)
C4—O2—C1—O1	−1.5 (6)	C16—O4—C12—O5	0.0 (7)
C7—C2—C3—C4	−0.7 (8)	C18—C13—C14—C15	−0.5 (7)
C2—C3—C4—C5	−0.2 (8)	C13—C14—C15—C16	−1.0 (7)
C2—C3—C4—O2	−178.6 (4)	C13—C14—C15—O5	−178.8 (4)
C1—O2—C4—C3	178.5 (6)	C12—O5—C15—C14	178.8 (6)
C1—O2—C4—C5	−0.1 (5)	C12—O5—C15—C16	0.8 (5)
C3—C4—C5—C6	−0.2 (9)	C14—C15—C16—O4	−179.0 (5)
O2—C4—C5—C6	178.5 (5)	O5—C15—C16—O4	−0.9 (6)
C3—C4—C5—O1	−177.1 (5)	C14—C15—C16—C17	2.9 (8)
O2—C4—C5—O1	1.6 (6)	O5—C15—C16—C17	−178.9 (5)
C1—O1—C5—C6	−179.3 (6)	C12—O4—C16—C15	0.5 (7)
C1—O1—C5—C4	−2.5 (7)	C12—O4—C16—C17	178.4 (6)
C4—C5—C6—C7	1.3 (9)	C15—C16—C17—C18	−3.2 (9)
O1—C5—C6—C7	177.6 (6)	O4—C16—C17—C18	179.1 (5)
C3—C2—C7—C6	1.7 (7)	C14—C13—C18—C17	0.1 (7)
C3—C2—C7—C8	−177.1 (5)	C14—C13—C18—C19	−178.2 (4)
C5—C6—C7—C2	−2.0 (8)	C16—C17—C18—C13	1.7 (8)
C5—C6—C7—C8	176.9 (5)	C16—C17—C18—C19	180.0 (5)
C2—C7—C8—C9	69.1 (5)	C13—C18—C19—C20	66.7 (5)
C6—C7—C8—C9	−109.7 (5)	C17—C18—C19—C20	−111.5 (5)
C10—N1—C9—C8	−109.7 (5)	C21—N2—C20—C19	−106.4 (5)
C7—C8—C9—N1	178.8 (3)	C18—C19—C20—N2	179.4 (3)
C9—N1—C10—O3	−1.6 (6)	C20—N2—C21—O6	−0.6 (6)
C9—N1—C10—C11	−179.3 (4)	C20—N2—C21—C22	179.3 (4)
O3—C10—C11—Cl2	178.4 (3)	O6—C21—C22—Cl1	177.3 (3)
N1—C10—C11—Cl2	−3.7 (6)	N2—C21—C22—Cl1	−2.6 (5)

### Hydrogen-bond geometry (Å, °)

**Table:**

*D*—H···*A*	*D*—H	H···*A*	*D*···*A*	*D*—H···*A*
N1—H1A···O3^i^	0.86	2.18	2.894 (4)	140
N2—H2B···O6^ii^	0.86	2.18	2.894 (4)	140

Symmetry codes: (i) *x*, *y*+1, *z*; (ii) *x*, *y*−1, *z*.

## References

[bb1] Allen, F. H., Kennard, O., Watson, D. G., Brammer, L., Orpen, A. G. & Taylor, R. (1987). *J. Chem. Soc. Perkin Trans. 2*, pp. S1–19.

[bb2] Bernhard, H. O. & Snieckus, V. (1971). *Tetrahedron*, **27**, 2091–2100.

[bb8] Flack, H. D. (1983). *Acta Cryst.* A**39**, 876–881.

[bb3] Higashi, T. (1995). *ABSCOR* Rigaku Corporation, Tokyo, Japan.

[bb4] Kuehne, M. E., Bornmann, W. G., Parsons, W. H., Spitzer, T. D., Blount, J. F. & Zubieta, J. (1988). *J. Org. Chem.* **53**, 3439–3450.

[bb5] Ma, C., Liu, S., Xin, L., Zhang, Q., Ding, K., Falck, J. R. & Shin, D. (2006). *Chem. Lett.* **35**, 1010–1011.

[bb6] Rigaku (2004). *RAPID-AUTO* Rigaku Corporation, Takyo, Japan.

[bb7] Sheldrick, G. M. (2008). *Acta Cryst.* A**64**, 112–122. 10.1107/S0108767307043930 18156677

